# The infraorbital vein: embryology, anatomy, histology, variations, and clinical relevance

**DOI:** 10.1007/s00276-026-03951-6

**Published:** 2026-07-24

**Authors:** Kazuaki Hasegawa, Yohei Takeshita, Shogo Kikuta, Seiichi Inoue, Mi-Sun Hur, Tatsuo Okui, R. Shane Tubbs, Joe Iwanaga

**Affiliations:** 1 Department of Maxillofacial Diagnostic and Surgical Science, Field of Oral and Maxillofacial Rehabilitation, Graduate School of Medical and Dental Sciences, Kagoshima University, Kagoshima, 890-8544 Japan; 2 Department of Oral and Maxillofacial Radiology, Faculty of Medicine, Dentistry and Pharmaceutical Sciences, Okayama University, Okayama, 700-8558 Japan; 3 Dental and Oral Medical Center, Kurume University School of Medicine, 67 Asahi-machi, Kurume, Fukuoka 830-0011 Japan; 4 Department of Neurosurgery, Tulane Center for Clinical Neurosciences, Tulane University School of Medicine, 131 S. Robertson St. Suite 1300, New Orleans, LA 70112 USA; 5 Division of Gross and Clinical Anatomy, Department of Anatomy, Kurume University School of Medicine, 67 Asahi-machi, Kurume, Fukuoka Japan; 6 Department of Orthopaedic Surgery, Kurume University School of Medicine, Fukuoka, Japan; 7 Department of Anatomy, Daegu Catholic University School of Medicine, 33, Duryugongwon-ro 17gil, Nam-gu, Daegu, Korea; 8 Department of Neurology, Clinical Neuroscience Research Center, Tulane University School of Medicine, New Orleans, LA USA; 9 Department of Structural & Cellular Biology, Tulane University School of Medicine, New Orleans, LA USA; 10 Department of Neurosurgery and Ochsner Neuroscience Institute, Ochsner Health System, New Orleans, LA USA; 11 Department of Anatomical Sciences, St. George’s University, St. George’s, Grenada; 12 Department of Surgery, Tulane University School of Medicine, New Orleans, LA USA; 13 University of Queensland, Brisbane, Australia

**Keywords:** Anatomy, Histology, Embryology, Face, Vein, Orbit

## Abstract

The infraorbital vein (IOV) is a small but clinically important component of the deep midfacial venous system, linking the infraorbital region, orbit, and infratemporal fossa. Despite its close association with the infraorbital nerve and artery, the IOV remains inconsistently described and systematically undercharacterized in the literature. This narrative review synthesizes current evidence on the embryology, anatomy, variations, and clinical relevance of the IOV to provide a clinically oriented framework. Embryologically, the IOV derives primarily from the primitive maxillary vein, with variable contributions from the orbitonasal venous channels, which explains its dual connections to the pterygoid venous plexus and the orbital venous system. Anatomically, it demonstrates considerable variability in presence, caliber, and spatial relationship within the infraorbital canal. The IOV may present as a single trunk, duplicated channels, or a plexiform network, with additional variation associated with accessory infraorbital foramina and emissary connections. Clinically, the IOV is relevant to orbital floor and zygomatic fracture repair, implant surgery, the spread of odontogenic infection, and esthetic procedures. Although often overlooked, the IOV represents a key component of a dynamic midface–orbit–skull base venous axis. Improved understanding of its anatomy and variation is essential for surgical planning and complication avoidance.

## Introduction

**Fig. 1:**
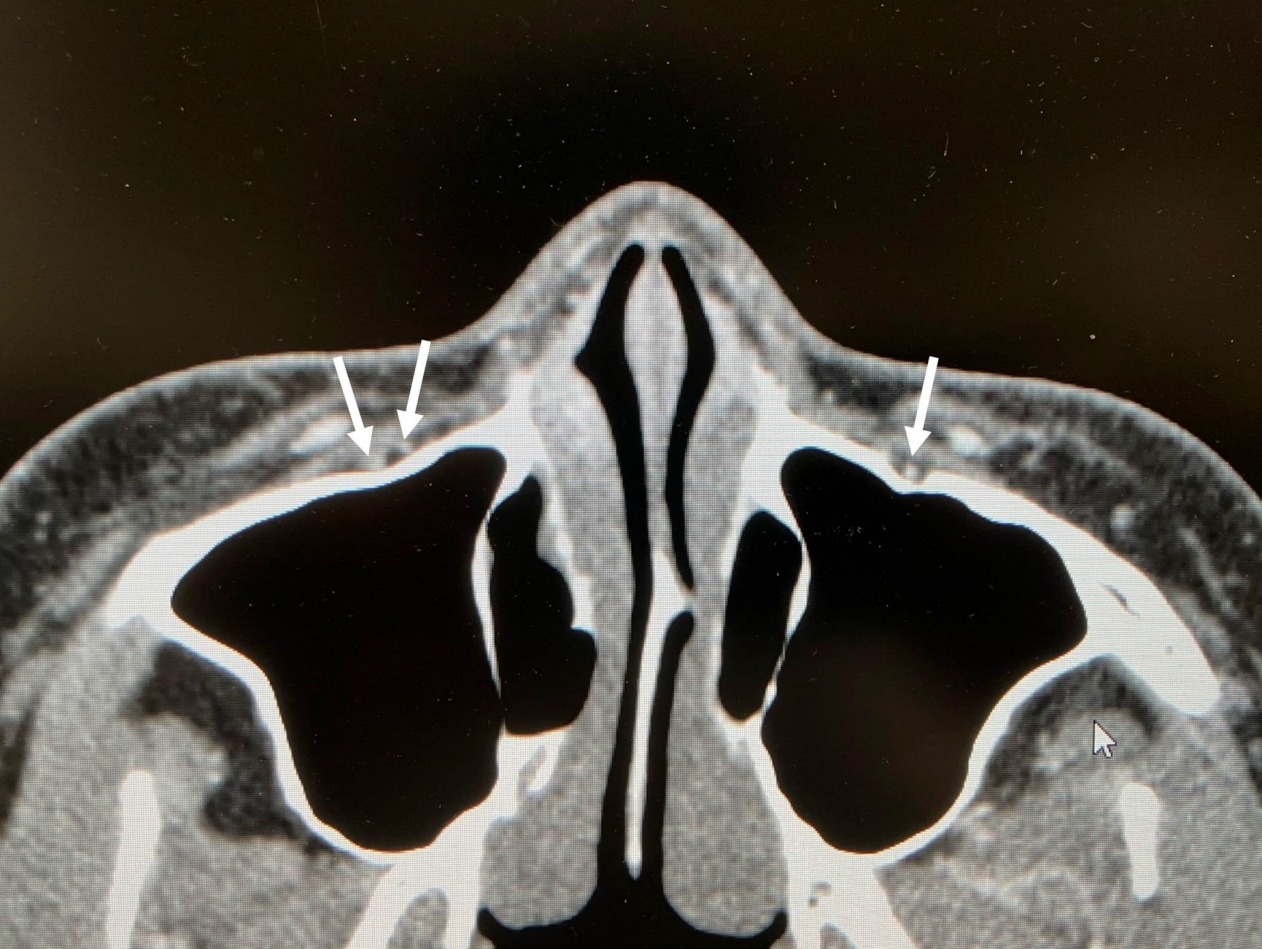
Axial contrast-enhanced CT image from a patient demonstrating the infraorbital vessels (arrows)

**Fig. 2:**
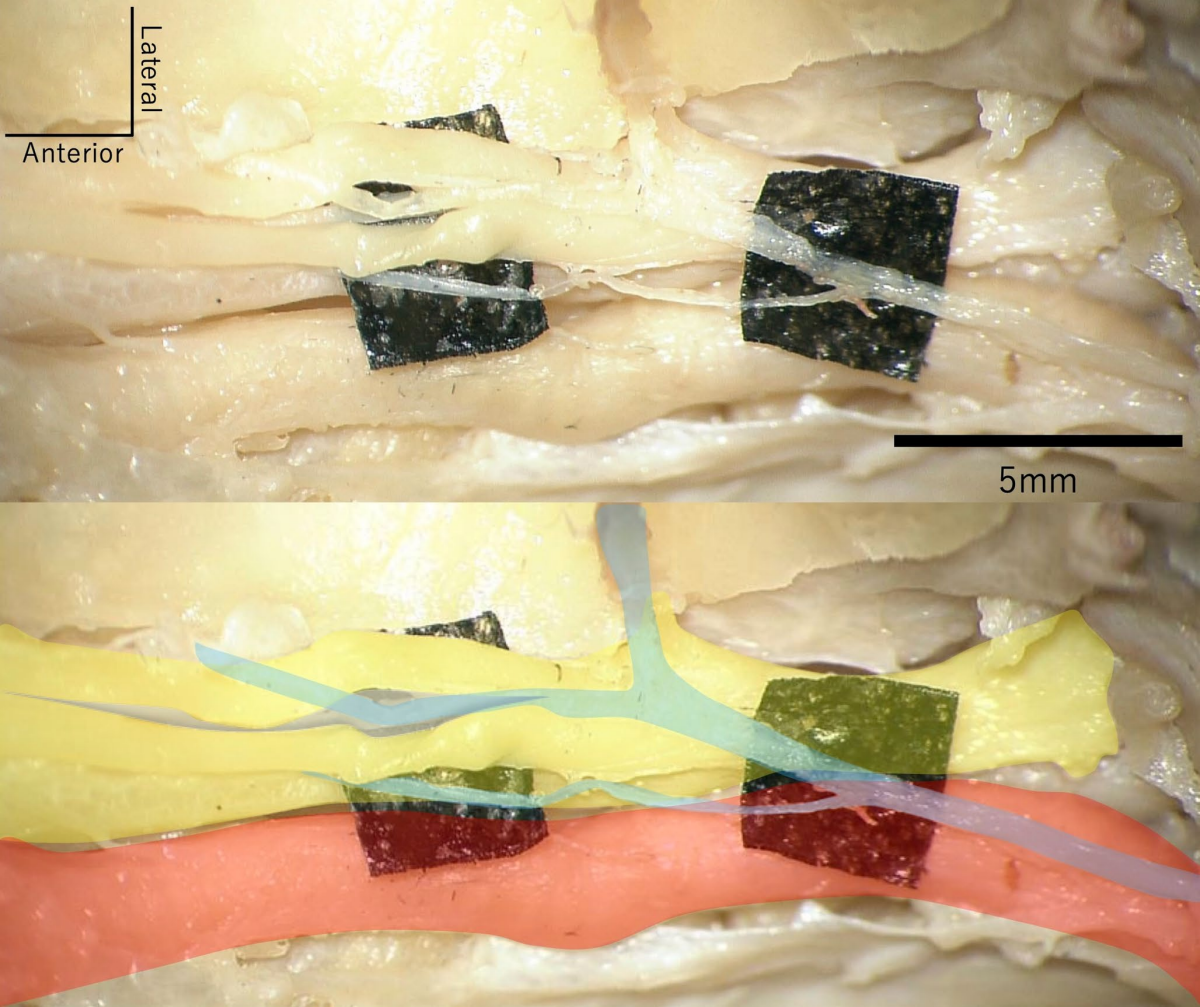
Inferior view of the infraorbital neurovascular bundles harvested from a human cadaver after removal of the inferior wall of the infraorbital canal. The nerve (yellow), artery (red), and vein (blue) were identified by microsurgical dissection under a surgical microscope, and the presumed veins were distinguished based on their anatomical features. Notably, the infraorbital vein demonstrates early branching within the canal, forming multiple small venous branches rather than a single trunk

The infraorbital vein (IOV) is an important yet understudied component of the venous network linking the midfacial soft tissues, the infraorbital canal, and the deep venous plexuses of the infratemporal fossa (Figs. 1 and 2). In contrast to the infraorbital artery and nerve—both of which have been the subject of numerous detailed anatomical and clinical studies—the venous counterpart is generally described only superficially, and its precise course and connections are often not elaborated in standard anatomical texts [35]. Some atlases depicted the IOV using drawings [20], but gross anatomical dissection, even with well-injected samples, often failed to clearly demonstrate the IOV [26]. This imbalance in the literature reflects broader inconsistencies in the depiction of deep midfacial venous pathways, with nomenclature and drainage patterns varying considerably across contemporary sources [10]. Within the orbit, venous anatomy shows marked interindividual variability, and small channels extending from the inferior ophthalmic vein toward the infraorbital region have been described [3, 19]. Venous return from the infraorbital canal drains posteriorly into the pterygoid venous plexus (PVP), establishing a deep outflow pathway from the midface to the infratemporal fossa [22, 25]. These findings indicate that the IOV participates in a variably developed venous connection between the midface, orbit, and PVP. For oral and maxillofacial surgeons, the infraorbital region represents a critical surgical corridor [5]. Procedures such as orbital floor reconstruction, zygomaticomaxillary complex fracture repair, orthognathic surgery, and esthetic interventions routinely expose the infraorbital groove and canal [5]. Injury to the vascular components of this bundle might lead to troublesome bleeding, postoperative hematoma, or, in rare cases, acute intraorbital hemorrhage and other orbital complications, although it is generally considered safe zone [5, 15, 31]. Because the infraorbital venous pathway drains toward the PVP, it also forms a potential route for the spread of odontogenic infection or septic thrombophlebitis into deeper craniofacial venous spaces, a mechanism highlighted in recent reviews of ophthalmic complications following dental procedures [36]. Understanding this anatomy is therefore essential for safe surgical access, accurate risk assessment, and the prevention of both hemorrhagic and infectious complications.

This review synthesizes current evidence regarding the embryology, anatomy, variations, and clinical relevance of the IOV. By integrating embryologic analyses and the infraorbital venous anatomy, we aim to provide a clinically oriented anatomical framework tailored to the needs of oral and maxillofacial surgeons.

For figures derived from cadaveric material, international consensus guidelines were followed [12, 14, 32]. This study was conducted as a narrative review and did not follow a formal systematic search protocol. Nevertheless, relevant literature was identified through structured searches of PubMed and Google Scholar using predefined keywords, and supplemented by manual screening of reference lists [13].

## Embryology

The venous anatomy of the midface and infratemporal fossa derives primarily from the primitive maxillary vein (PMV), which constitutes the principal drainage pathway of the maxillary prominence and the ventral orbitonasal region during early embryogenesis [25]. The PMV develops in close association with the maxillary nerve (V2), forming the embryologic basis for the adult topographic coupling of the infraorbital venous, arterial, and neural structures. As craniofacial development progresses, the PMV redistributes into a superficial component contributing to the facial vein and a deep component forming the maxillary vein and PVP [25]. In parallel, the stapedial–external carotid arterial system gives rise to the infraorbital artery, which comes to accompany V2 toward the developing infraorbital groove and canal, representing a parallel arterial development that establishes the topographic neurovascular bundle of the infraorbital region [2]. The osteogenic formation of the infraorbital canal subsequently encloses this neurovascular bundle. Primitive orbitonasal venous channels, as described in embryological studies of ophthalmic and cavernous sinus development, connect the ventral orbit to the maxillary prominence during early morphogenesis [33]. These channels persist variably and provide a developmental explanation for the orbital tributaries that frequently enter the proximal infraorbital venous pathway in the adult. Taken together, the IOV may present an anterior tributary of the developing maxillary–pterygoid venous system that maintains variable communications with orbitonasal venous channels, while a partial dual developmental contribution from orbitonasal and maxillary venous systems remains plausible. From a developmental and topographic perspective, the IOV may be regarded as an interface between the deep maxillary–pterygoid venous network and the orbitonasal venous plexus, which accounts for its variable orbital connections and consistent drainage into the PVP and maxillary vein. Variability in diameter, dominance, and orbital communication may reflect differential persistence or regression of primitive orbitonasal and maxillary venous channels.

### Anatomy

The IOV, although small, generally corresponds to the infraorbital artery and is a tributary of the PVP, and through it, of the retromandibular vein [39]. It receives tributaries from structures located near the floor of the orbit and communicates with the inferior ophthalmic vein [27]. The inferior palpebral veins, typically two or three, descend from the lower eyelid and establish communication with the IOV [7]. The IOV also communicates with the facial vein, descending palatine vein, pharyngeal veins, vein of the pterygoid canal, and buccal veins [1]. Functionally, it drains the midface, including the lower eyelid, lateral nose, and upper lip, ultimately communicating with the PVP [21].

Anatomically, the IOV accompanies the infraorbital nerve and artery within the infraorbital canal as part of the V2 neurovascular bundle, with possible lymphatic components also present in the canal [38]. It courses along the floor of the orbit and passes posteriorly through the inferior orbital fissure to join the PVP [30]. Upon exiting the infraorbital canal, it turns posteriorly and drains into the PVP, which subsequently converges into the maxillary vein, forming the principal deep midfacial venous drainage pathway [22, 25, 35].

Within the infraorbital canal, the relative arrangement of the neurovascular structures is variable. A commonly described configuration places the artery superiorly, the nerve centrally, and the vein inferiorly or inferomedially [16, 18, 34]. However, variations exist, with reports describing alternative positional relationships or even the absence of a distinct vein. For example, Czermak (1895) illustrated a vein medial to the nerve, whereas other observations have found the artery positioned superior and medial to the nerve with no identifiable vein. Historical accounts also reflect inconsistency: Stanculeanu (1902) described the presence of the IOV, whereas Sesemann failed to identify it, and Festal considered it exceptional [38].

The proximal portion of the IOV receives small venous tributaries from the orbital floor, which converge toward the infraorbital groove [3, 33]. These tributaries vary widely in number and caliber, and a distinct vein at the infraorbital foramen is identifiable in only approximately half of adult specimens [16]. In a minority of individuals, accessory drainage pathways toward the facial or angular veins are observed, reflecting persistence of superficial venous channels [10].

## Accessory foramina and IOV

Beyond these soft-tissue communications, accessory openings of the infraorbital region introduce an additional layer of anatomical variability. Multiple anatomical and radiologic studies have reported the presence of an accessory infraorbital foramen (AIOF) in approximately 10–20% of individuals, with population-level frequencies ranging from 10% to 18% in cadaveric, dry-skull, and CT-based investigations [9, 24, 29]. Although the presence of an AIOF suggests a potential additional neurovascular channel, a bony foramen alone does not confirm the presence of a venous structure, as most studies have focused on osseous morphology rather than soft tissue contents. Only a limited number of cadaveric studies have examined accessory infraorbital foramina and their associated nerves [11]. Accordingly, AIOF frequency should be interpreted as a marker of potential accessory pathways, rather than definitive evidence of an accessory vein.

Nevertheless, the consistent 10–20% prevalence across populations suggests that anatomical variation at the infraorbital exit is common, and this may partially account for the diversity of drainage patterns and emissary connections described in the adult infraorbital venous system.

## Histology

**Fig. 3:**
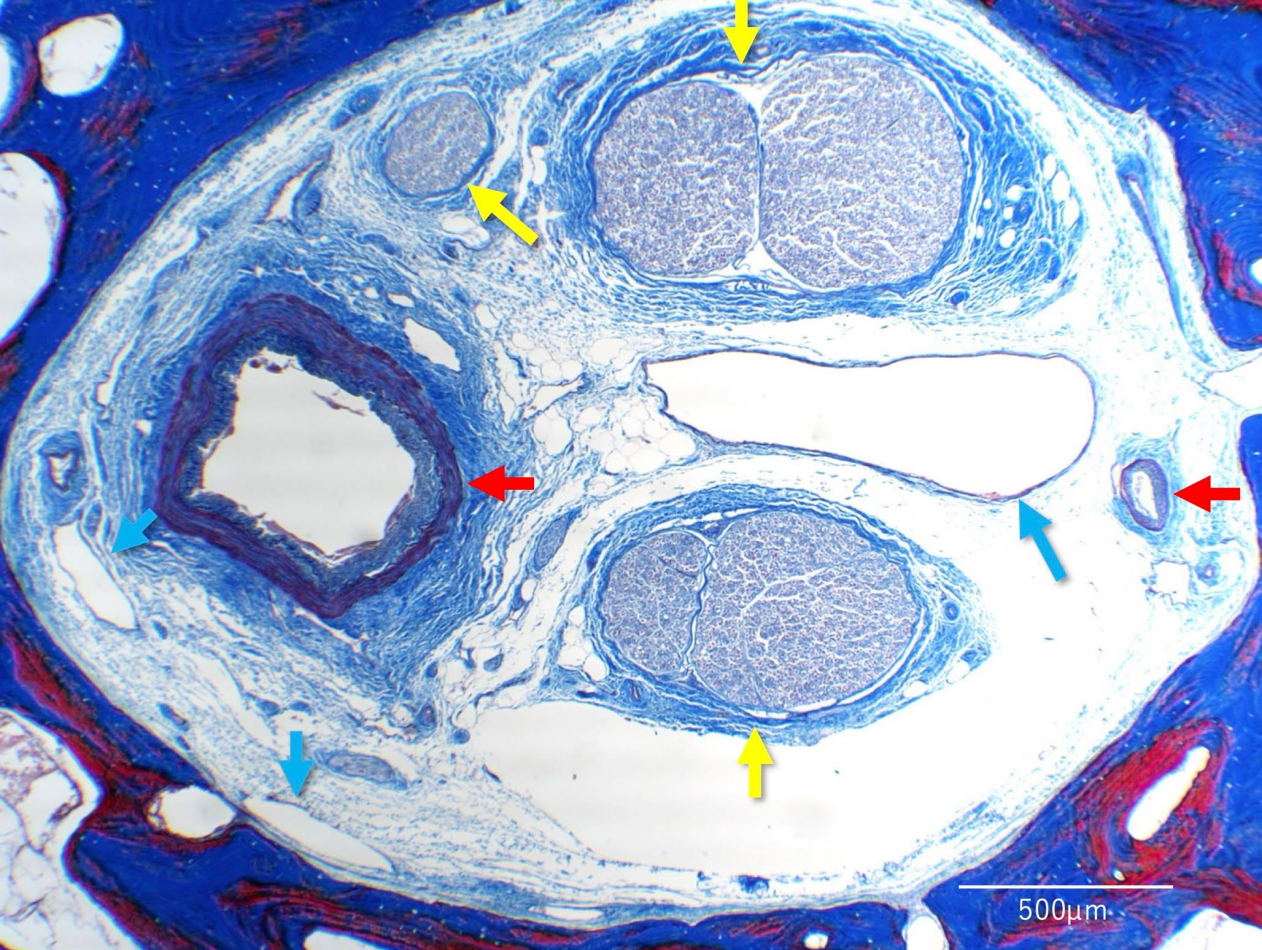
Cross sectional histological image of the infraorbital canal obtained from a human cadaver and stained with Masson’s trichrome. Note that the canal contains infraorbital nerves (yellow arrows), infraorbital arteries (red arrows), and infraorbital veins (blue arrows). The veins appear as multiple irregularly distributed small branches throughout the infraorbital canal

There are very few studies that have investigated the IOV from a histological perspective (Fig. 3). Polo et al. (2018) examined histological sections of the infraorbital canal; however, they provided little discussion regarding the venous structures. Similarly, Muchlinski (2008) investigated the infraorbital canal in mammals and presented coronal sections of the canal, but references to the vein were minimal. Thus, the IOV has not been a major focus of detailed histological investigation to date.　Further histological investigation is required to clarify the structural characteristics of the venous wall and its relationship to surrounding neurovascular components.

## Clinical relevance

### Orbital floor and zygomatic fractures

Orbital floor and zygomatic fractures are common indications for surgical intervention, and the infraorbital canal is often directly involved in these injuries [15]. Dissection along the orbital floor requires elevation of the periosteum and exposure of the infraorbital groove and canal, where the infraorbital artery and vein can be a source of intraoperative bleeding [5, 35]. A case report of acute intraorbital hemorrhage following reduction of a displaced zygomatic fracture illustrates how venous injury in this region can precipitate an orbital compartment syndrome with visual compromise [31]. The authors suggested that damage to venous channels at the orbital floor or infraorbital fissure was the most likely source of bleeding in that case [31]. Others reported cases of orbital and zygomatic fractures followed by hemorrhage although it is not common [4, 17]. Knowledge of the extensive venous communications between the IOV, facial vein, and PVP highlights the potential for significant venous bleeding from the infratemporal region when the infraorbital canal is injured [22]. For surgeons, this underscores the importance of careful dissection, hemostatic preparation, and prompt recognition of signs of orbital compartment syndrome during fracture repair [15]. In our observations, the infraorbital vein demonstrated a plexiform configuration with multiple small branches already present within the infraorbital canal, which may further increase the risk of diffuse venous injury and bleeding in this region.

### Zygomatic implant surgery

Zygomatic implant placement represents another clinically relevant context in which the infraorbital foramen/canal may be implicated. Ocular and intraorbital complications, including diplopia, orbital hemorrhage, and visual disturbances, have been reported following zygomatic implant procedures, likely related to the close anatomical relationship between the implant trajectory, the orbital floor, and the infraorbital neurovascular bundle [36, 37]. Given that the IOV communicates variably with orbital venous channels and ultimately drains into the PVP, vascular injury or compression during implant osteotomy may contribute to venous congestion, intraorbital hemorrhage, or indirect orbital complications. Although no studies have specifically examined the role of the IOV in zygomatic implant-related complications, its variable anatomy, including differences in diameter, plexiform morphology, and orbital communications, may be relevant and merits further investigation.

## Odontogenic infection and cavernous sinus involvement

Odontogenic infections of maxillary origin can, in rare cases, progress to orbital cellulitis or cavernous sinus thrombosis [23, 28, 36, 40]. A reported case of dental infection presenting with parapharyngeal abscess and contralateral orbital cellulitis highlights the complexity of venous routes between the maxilla and the orbit [6, 23]. Anatomical studies indicate that the PVP receives drainage from maxillary and deep facial territories and communicates with the cavernous sinus via emissary veins [22]. Given that the IOV connects midfacial tissues to the PVP, this pathway provides a plausible conduit for retrograde spread of septic thrombophlebitis from dental infections to intracranial venous sinuses [33]. Clinically, infraorbital swelling and pain from dental sepsis should prompt careful assessment for orbital signs, and imaging is warranted when deep-space involvement is suspected [36]. Early and aggressive management, including drainage and intravenous antibiotics, is critical to prevent progression to cavernous sinus thrombosis [6].

## Esthetic procedures in the infraorbital and malar region

The infraorbital region is a frequent target for dermal fillers and other aesthetic injections. Near-infrared imaging studies have shown that superficial veins in the periorbital and malar regions can be visualized and, to some extent, avoided using vein-mapping devices [8]. However, deeper veins, such as the IOV, are not reliably visualized with these techniques and therefore still pose a potential risk of venous injury or compromise [8]. Anatomical reviews emphasize that the IOV acts as a major deep outflow tract from infraorbital soft tissues, which means that filler injection into this region should be performed with attention to depth, plane, and injection pressure [10]. For surgeons and esthetic practitioners, understanding the three-dimensional course of the IOV supports the use of blunt cannulas, controlled injection, and safe zones that minimize the likelihood of damaging deep venous structures [35].

## Conclusions

Current knowledge of the infraorbital vein is derived from a combination of embryologic analyses, anatomical dissections, histological images, angiographic studies, and clinical observations [16, 22, 33]. These data consistently support the concept of the IOV as an important deep venous pathway connecting the midface, inferior orbit, and PVP [10]. From a clinical standpoint, this pathway is relevant to orbital floor and ZMC trauma, odontogenic infections, and complications of esthetic procedures [8, 15, 31, 36]. Despite its importance, the IOV has received far less attention than its neural and arterial counterparts in both clinical and anatomical literature [10]. Future research could include selective venographic studies of the infraorbital venous system, high-resolution imaging correlating bony variants with venous patterns and detailed cadaveric injection work focusing specifically on the IOV [25]. Such studies would help refine vessel-specific anatomy and provide practical information for surgeons operating in the infraorbital corridor [35]. For oral and maxillofacial surgeons, recognizing the infraorbital vein as part of a broader midface–orbit–skull base venous axis enables more informed surgical planning and risk assessment. A precise understanding of its embryology, course, variations, and clinical implications can contribute directly to safer and more effective management of midfacial and orbital conditions. Future developmental studies are needed to clarify the embryologic origin and maturation of the infraorbital vein.

## Data Availability

No datasets were generated or analysed during the current study.
